# A retrospective cohort analysis leveraging augmented intelligence to characterize long COVID in the electronic health record: A precision medicine framework

**DOI:** 10.1371/journal.pdig.0000301

**Published:** 2023-07-25

**Authors:** Zachary H. Strasser, Arianna Dagliati, Zahra Shakeri Hossein Abad, Jeffrey G. Klann, Kavishwar B. Wagholikar, Rebecca Mesa, Shyam Visweswaran, Michele Morris, Yuan Luo, Darren W. Henderson, Malarkodi Jebathilagam Samayamuthu, The Consortium for Clinical Characterization of COVID-19 by EHR (4CE), Gilbert S. Omenn, Zongqi Xia, John H. Holmes, Hossein Estiri, Shawn N. Murphy

**Affiliations:** 1 Department of Medicine, Massachusetts General Hospital, Boston, Massachusetts, United States of America; 2 Department of Electrical Computer and Biomedical Engineering, University of Pavia, Pavia, Italy; 3 Institute of Health Policy, Management and Evaluation, Dalla Lana School of Public Health, University of Toronto, Toronto, Canada; 4 Department of Biomedical Informatics, University of Pittsburgh, Pittsburgh, Pennsylvania, United States; 5 Department of Preventive Medicine, Northwestern University, Chicago, Illinois, United States of America; 6 Center for Clinical and Translation Science, University of Kentucky, Lexington, Kentucky, United States of America; 7 Department of Biomedical Informatics, Harvard Medical School, Boston, Massachusetts, United States of America; 8 Dept of Computational Medicine & Bioinformatics, Internal Medicine, Human Genetics, and School of Public Health, University of Michigan, Ann Arbor, Michigan, United States of America; 9 Department of Neurology, University of Pittsburgh, Pittsburgh, Pennsylvania, United States of America; 10 Department of Biostatistics, Epidemiology, and Informatics; Institute for Biomedical Informatics, University of Pennsylvania Perelman School of Medicine, Philadelphia, Pennsylvania, United States of America; 11 Department of Neurology, Massachusetts General Hospital, Boston, Massachusetts, United States of America; Tsinghua University, CHINA

## Abstract

Physical and psychological symptoms lasting months following an acute COVID-19 infection are now recognized as post-acute sequelae of COVID-19 (PASC). Accurate tools for identifying such patients could enhance screening capabilities for the recruitment for clinical trials, improve the reliability of disease estimates, and allow for more accurate downstream cohort analysis. In this retrospective cohort study, we analyzed the EHR of hospitalized COVID-19 patients across three healthcare systems to develop a pipeline for better identifying patients with persistent PASC symptoms (dyspnea, fatigue, or joint pain) after their SARS-CoV-2 infection. We implemented distributed representation learning powered by the Machine Learning for modeling Health Outcomes (MLHO) to identify novel EHR features that could suggest PASC symptoms outside of typical diagnosis codes. MLHO applies an entropy-based feature selection and boosting algorithms for representation mining. These improved definitions were then used for estimating PASC among hospitalized patients. 30,422 hospitalized patients were diagnosed with COVID-19 across three healthcare systems between March 13, 2020 and February 28, 2021. The mean age of the population was 62.3 years (SD, 21.0 years) and 15,124 (49.7%) were female. We implemented the distributed representation learning technique to augment PASC definitions. These definitions were found to have positive predictive values of 0.73, 0.74, and 0.91 for dyspnea, fatigue, and joint pain, respectively. We estimated that 25 percent (CI 95%: 6–48), 11 percent (CI 95%: 6–15), and 13 percent (CI 95%: 8–17) of hospitalized COVID-19 patients will have dyspnea, fatigue, and joint pain, respectively, 3 months or longer after a COVID-19 diagnosis. We present a validated framework for screening and identifying patients with PASC in the EHR and then use the tool to estimate its prevalence among hospitalized COVID-19 patients.

## Author summary

Analyzing long COVID using the healthcare system’s electronic health records presents unique challenges due to variable coding practices by healthcare providers and medical coders. For instance, different providers may emphasize different aspects of a patient’s condition such as shortness of breath versus the underlying cause of the symptom (i.e., COVID-19, congestive heart failure or chronic obstructive pulmonary disease). Additionally, some health records may only hint at new or persistent symptoms through a new prescription, a procedure, or a laboratory order. This complexity was heightened prior to the introduction of the long COVID billing code since there was not a clear consensus for how to code patients with ongoing symptoms. Our study utilized a novel representation learning approach to navigate these challenges. We built models using diverse electronic health record data (diagnosis, medication, procedure, and laboratory orders) gathered from several hospital systems to better identify patients showing potential signs of long COVID. We validated the accuracy of our models by manual patient chart reviews. Using this method, we obtained estimates of hospitalized COVID-19 patients exhibiting dyspnea, fatigue, or joint pain three months post-hospitalization. Our augmented definitions can be used to identify potential long COVID patients from the structured data in the electronic health records.

## Introduction

Persistent physical symptoms lasting months following an acute COVID-19 infection are well known and now widely documented [1–5]. Psychological or cognitive complaints have also been reported during recovery from the SARS-CoV-2 infection [6–8]. These patients have been collectively referred to as having long COVID, post-acute COVID-19 syndrome (PACS), or post-acute sequelae of SARS-CoV-2 (PASC). While the exact definition continues to evolve, there is general agreement that it refers to symptoms that persist or relapse at least 3 months from the onset of acute infection, have an impact on the patient’s life, and are not explained by an alternative cause [9,10].

Several large epidemiology studies have now been published that attempt to quantify the prevalence of long COVID and characterize the etiology [11–16]. While valuable, the insights from these studies rely primarily on analyses of the diagnosis codes from the electronic health records. There are several limitations when exclusively using diagnosis codes for identifying specific patients. This is because the diagnosis codes are not meant to be research quality data, but are instead assigned through the transactional interaction between the healthcare system and patient. The diagnoses only indirectly represent an individual’s actual health [17]. Previous studies have found variable rates of sensitivity and specificity for diagnosis codes to accurately describe symptoms and disease [18–21]. This makes using the diagnosis codes from electronic health records challenging for studying long COVID. Many patients that have a specific symptom may not have it documented as researchers expect, and those that have the diagnosis may not actually have the symptom. Adding to this complexity is that the “U09.9 long COVID” code itself was not introduced until late in the pandemic. If only the “U09.9 long COVID” is used for identification, patients with onset of long COVID early in the pandemic would be missed. There is also growing evidence that there is a spectrum of long COVID [22–24]. The long COVID diagnosis code does not differentiate among the long COVID symptom types. For each of these reasons, a data driven process for selecting codes that is validated for identifying long COVID is needed.

This study proposes a framework for developing enhanced definitions for detecting long COVID. We focused on three common and well-known symptoms of long COVID: dyspnea, fatigue, and joint pain [1–5,25,26]. We implemented an augmented intelligence strategy that combines machine learning methodology with clinical knowledge to improve the groups of diagnosis codes representing a specific symptom with additional multi-modal data from the EHR. Then we assessed the quality of the enhanced definition by reviewing clinical notes to estimate the positive predictive value of the new definition. Based on this assessment we then estimated the number of patients previously hospitalized with COVID-19 who are likely to develop long COVID symptoms.

## Methods

**Fig 1:**
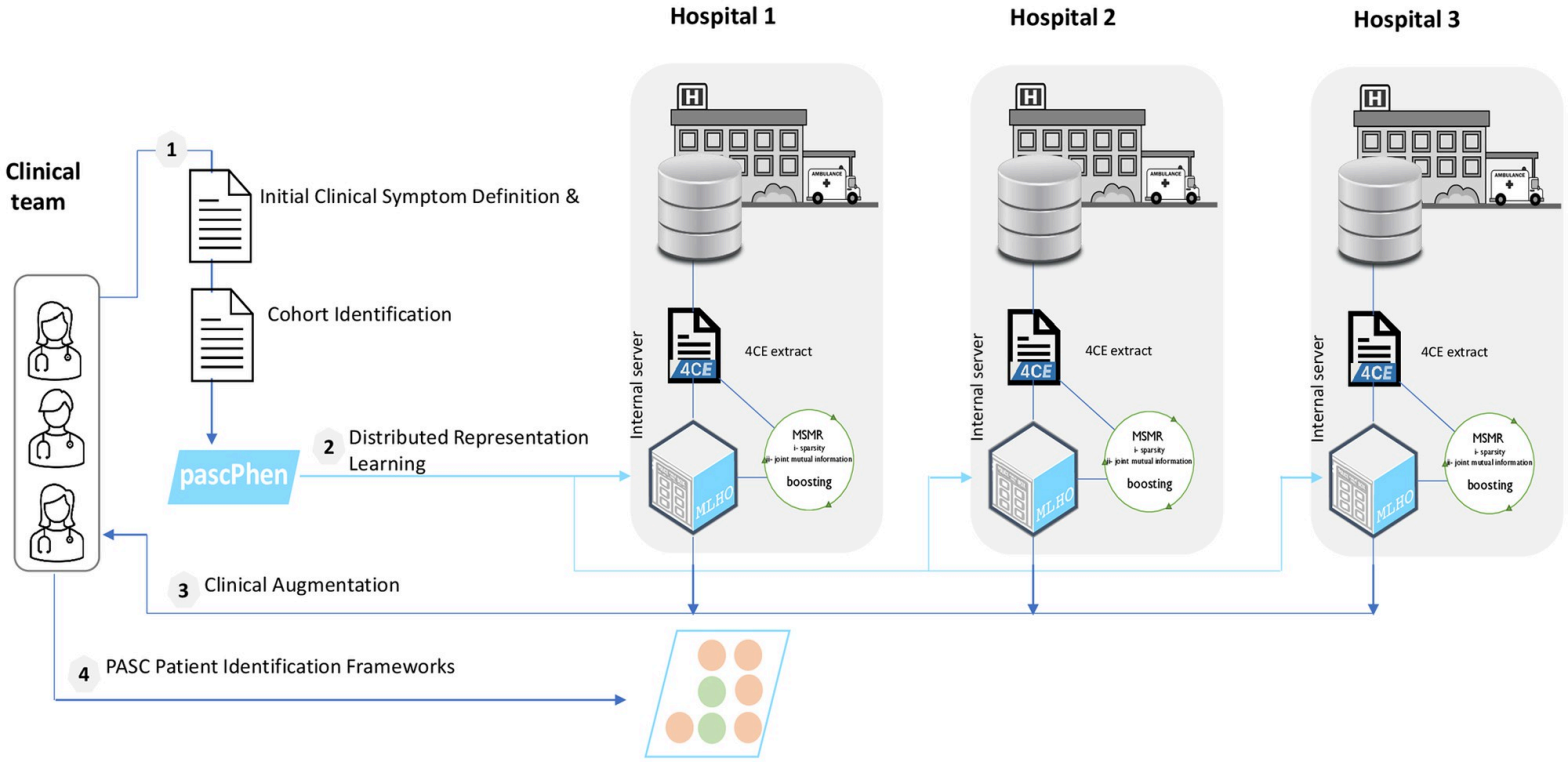
The augmented intelligence framework for identifying long COVID patients.

In order to develop our PASC definitions, we utilized EHR data from three academic healthcare systems in the United States that participate in the 4CE consortium [27–29]. Each contributing institution received institutional review board approval for aggregate data sharing. No patient level data were shared outside of the respective institution. We employed a validated machine learning framework for modeling evolving phenotypes, MLHO [30], with proven utility in studying long COVID [31], to enrich an expert-curated definition for each of the three PASC problems (dyspnea, fatigue, joint pain), through a distributed representation learning process (Fig 1). We then evaluated the MLHO-produced representation based on clinical expertise to develop and validate the framework for providing population-level estimates. Our study followed the Strengthening the Reporting of Observational Studies in Epidemiology (STROBE) reporting guideline.

### Data set

We leveraged the data and network in the international Consortium for Clinical Characterization of COVID-19 by EHR (4CE) [27]. Members of the consortium use Integrating Biology and the Bedside (i2b2) or Observational Medical Outcomes Partnership (OMOP) platforms to map their data to a common data model. The data are harmonized locally and then shared in an aggregated form for analysis and visualization. Three hospital systems (Mass General Brigham, the University of Kentucky, and the University of Pittsburgh Medical Center) collaborated through the 4CE network to create local data sets for analyzing long COVID. The inclusion criteria were that patients needed a positive SARS-CoV-2 polymerase chase reaction (PCR) test for the first time 7 days before to 14 days after the time of hospitalization. Additionally, the COVID-19 hospitalization needed to occur between March 13, 2020 and February 28, 2021. Both adults and children were considered. There were no exclusion criteria. Each center then extracted its own EHR data elements, including ICD-10 diagnosis codes and pre-specified laboratory tests, medications, and procedure codes (S1 Table) for local analysis.

ICD-10 codes from 1 year to 14 days before the COVID admission were grouped with Elixhauser Comorbidity Software Refined using the R package “comorbidity” to determine patient comorbidities. [32] Several of the Elixhauser comorbidities were further consolidated based on clinical similarity (S2 Table).

### Initial clinical symptom definition and cohort identification

First, we grouped *International Classification of Diseases*, *Tenth Revision* (ICD-10) codes that best match the symptom of interest. For example, in the case of dyspnea, the definition included all diagnosis codes within the R06 group, which represents “Abnormalities of Breathing”. Previous COVID-19 studies used similar groupings of ICD-10 codes to represent specific PASC symptoms [11]. These initial data elements used to define the symptoms of interest are referred to as the *core* data elements throughout the manuscript. A complete list of the core data elements for each of the three symptoms can be found in S3 Table.

### Distributed representation learning and clinical augmentation

Next, we used a Machine Learning (ML) approach to identify additional structured data elements that could signal the presence of one of the three symptoms, but were not included as one of the original core features. These newly identified features are referred to as the *augmented features*. To determine these augmented features each of the three academic sites used their hospitalized COVID-19 patients to develop three different training sets. Each training set focused on a specific symptom. Patients were labeled as having the symptom if the core data element appeared for the first time at least 90 days after a COVID-19 hospitalization, with a look back period of one year before hospitalization. Patients were labeled as a negative case if they had a follow-up appointment at least 90 days after hospitalization and did not have a new core data element. This created three training sets per hospital with positive and negative cases.

We then implemented a previously described pipeline for identifying additional features that discriminate between the positive and negative patients in each training sample [31]. First, the core features used to identify the positive cases were removed from the training set. All other diagnosis codes, laboratory orders, medication orders, and procedure codes were considered possible features for discriminating between positive and negative patients. Then features were selected using a sparsity screen (required 2% prevalence), computation of joint mutual information, and boosting to identify those with highest association. Then a 5-fold cross validation is performed (80–20 train-test split) to develop a confidence score based on the number of times a particular feature is used in the model to identify a positive case (S2 Text).

The clinical team then reviewed the EHR features identified at all three sites (S4 Table) to assess if they were clinically meaningful (and therefore likely generalizable) and whether their incorporation into the original definition for each PASC phenotype would potentially result in an enrichment of the definition. To standardize and facilitate this process, we developed categories that could explain the underlying association between the identified data elements and the PASC symptom. All of the categories deemed likely to enhance the initial definitions were incorporated into a new definition, which were referred to as the augmented definition. This augmented definition includes both the original core features and the new augmented features. In contrast, the original definitions only have the core features.

### Validation of proposed model

The augmented definitions were then implemented in one of the three healthcare systems to identify the SARS-CoV-2 hospitalized patients most likely to have the specified persistent symptoms three months after the index date (positive PCR test). These patients were subdivided into four distinct groups (S1 Fig) for chart review and validation. Group 1 included those patients with both the core and augmented features. Group 2 included those with exclusively the core features but not the augmented features. Group 3 included those exclusively with the augmented features but not the core features. Group 4 was identified without any of the core or augmented features. Charts were then randomly sampled from each of the groups for review. The charts were examined by clinicians for descriptive language in the clinical note that would confirm the presence of the specific symptom after the COVID-19 diagnosis.

### Analysis of long COVID subgroups

Based on the sampled charts for each of the groups a positive predictive value for each of the feature sets was determined. Then a confidence interval was applied to the local population with the standard formula:

p±1.96×√((1/n)×p×(1-p)×(N-n)/(N-1))

where *p* is the sample proportion, 1.96 is the critical value of the normal distribution for a confidence level of 95%, *n* is the sample size and *N* is the population size. The groups were assumed to be independent of each other and were then summed to determine the estimated proportion of hospitalized COVID-19 patients with the specific persistent symptoms at 3 months with 95% confidence intervals.

Finally, the augmented definition was used to identify patients with likely long COVID. The identified patients in each of the symptom clusters were examined based on a variety of characteristics including age and comorbidities prior to COVID-19 diagnosis.

## Results

A total of 30,422 patients were diagnosed with COVID-19 across the three healthcare systems between March 13, 2020 and February 28, 2021. The mean age of the population was 62.3 (SD, 21.04). 15,124 (49.7%) were female. Many of the patients had at least one comorbidity documented in the EHR prior to the index date of positive PCR test for SARS-CoV-2. This included 44.4% with hypertension, 24.4% with diabetes, and 19.8% with a chronic pulmonary disease. For a complete list of demographics and comorbidities, as well as admission quarter, see Table 1.

**Table 1:** Characteristics of patients from the three healthcare systems.

	Patient No. (%)			
	Total (n = 30,422)	Hospital System 1 (n = 8344)	Hospital System 2 (n = 1952)	Hospital System 3 (n = 20,126)
**Mean Age, years (SD)**	62.3 (21.04)	61.2 (19.6)	51.7 (22.0)	63.8 (21.2)
**Median Age, years (IQR)**	-	63 [48–76]	56 (36–68)	68 (54–79)
**Era of Diagnosis**				
**2020Q1**	372 (1.2)	305 (3.65)	-	67 (0.33)
**2020Q2**	2777 (9.1)	2,330 (27.9)	44 (2.3)	403 (2.0)
**2020Q3**	1229 (4.0)	329 (3.94)	80 (4.1)	820 (4.1)
**2020Q4**	6605 (21.7)	1,625 (19.5)	206 (10.6)	4774 (23.7)
**2021Q1**	4481 (14.7)	1,576 (18.9)	200 (10.2)	2705 (13.4)
**2021Q2**	2147 (7.1)	559 (6.70)	118 (6.0)	1470 (7.3)
**2021Q3**	3260 (10.7)	551 (6.60)	745 (38.1)	1964 (9.8)
**2021Q4**	6251 (20.5)	1,011 (12.1)	559 (28.6)	4681 (23.3)
**2022Q1**	3300 (10.8)	58 (.70)	-	3242 (161.)
**Sex**				
**Men**	15,296 (50.3)	4,279 (51.3)	899 (46.1)	10,118 (50.3)
**Women**	15,124 (49.7)	4,065 (48.7)	1051 (53.8)	10,008 (49.7)
**Comorbidities**				
**Hypertension**	13,504 (44.4)	3215 (38.5)	419 (21.5)	9870 (49.0)
**Diabetes**	7410 (24.4)	1738 (20.8)	260 (13.3)	5412 (26.9)
**Cardiovascular diseases**	8679 (28.5)	2549 (30.5)	288 (14.8)	5842 (29.0)
**Neurological diseases**	3093 (10.2)	738 (8.8)	121 (6.2)	2234 (11.1)
**Malignant tumor**	734 (2.4)	270 (3.2)	53 (2.7)	411 (2.0)
**Chronic pulmonary disease**	6029 (19.8)	1303 (15.6)	192 (9.8)	4534 (22.52)
**Chronic kidney disease**	4953 (16.2)	1304 (15.6)	153 (7.8)	3496 (17.4)

Data are n (%), n/N (%), or median (IQR). The differing denominators indicate missing data. Within the category sex, the category “other” was designated at some hospitals, but the results were censored due to the number being low.

The core definitions for joint pain, fatigue, and dyspnea were each modeled in the three contributing healthcare systems. When the original features used to define dyspnea were excluded from the feature set, MHLO was still able to identify patients labeled as having dyspnea using the remaining features, reaching high accuracy with AUC’s ranging from 0.81 to 0.91 across the three hospital systems. For fatigue, the AUC varied from 0.84 to 0.90 between the three hospital systems. For joint pain, the AUC ranged from 0.78 to 0.84 (S5 Table).

**Fig 2:**
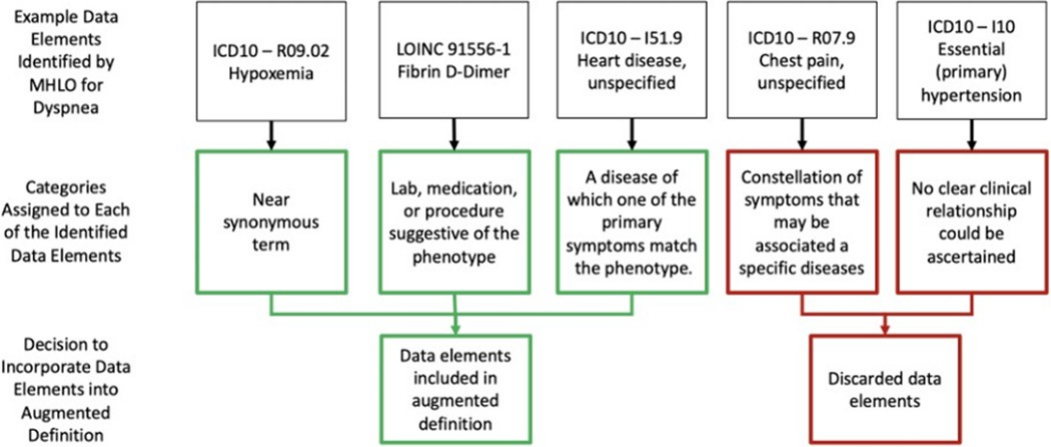
The strategy for deciding whether an identified MHLO data elements should be included in the augmented definition using dyspnea as an example of PASC phenotype.

After reviewing the newly identified pool of EHR features, the clinical team developed five categories to describe the association between the identified data elements by MHLO and the defined phenotype. The first category included data elements that were near synonyms for the phenotype but were not originally included in the definition. For example, in the case of dyspnea, the ICD code for hypoxemia was identified by MHLO. The second category comprised data elements as alternative to diagnosis codes such as laboratory tests, medications, or procedures that could suggest a particular phenotype was being investigated or treated by the clinicians. In the case of dyspnea, this could be a Logical Observation Identifiers Names and Codes (LOINC) code for the order of the D-dimer test, which could imply there was a concern for shortness of breath. The third category contained data elements that represent a disease for which the symptom could be the specific phenotype that was being defined. In the case of dyspnea, MHLO identified EHR data elements such as the ICD code for heart disease. Any of these categories could suggest the underlying symptom. The final two categories of EHR data elements identified by MHLO had unclear relationships with the underlying phenotype and were less helpful for augmenting the original definition. The fourth category included symptoms that may have some association with the phenotype (such as chest pain in the setting of shortness of breath) but were not synonyms with the specific symptom. The fifth category included data elements for which the relationship could not be understood clinically and may be related to utilization or another artifact in the EHR. (see Fig 2).

**Fig 3:**
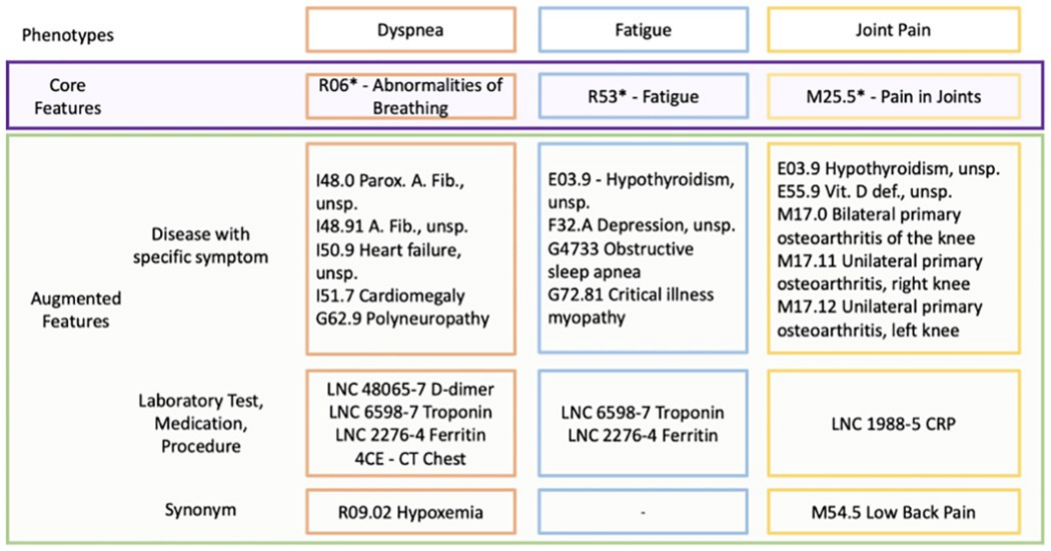
Augmented feature set for identifying persistent dyspnea, fatigue, and joint pain.

Each of the features identified by MHLO were classified into the specific category that best describe its relationship with the symptom. Those features that fit into one of the three categories thought likely to enhance the original phenotype, were then included in the augmented definition. The core and augmented features for each of the three phenotypes are shown in Fig 3.

After the augmented feature set was defined, it was then applied to one of the three healthcare systems (healthcare system 1), for which 5.0% of the hospitalized population had both the core and augmented data elements for dyspnea. 3.2% had exclusively at least one core feature and 17% had exclusively at least one of the augmented features for dyspnea. 75.3% had neither of the features. (Table 2). For fatigue, 6.0% had both the core and augmented features, 1.8% had exclusively the core feature, and 23.5% had exclusively one of the augmented features. 68.7% had none of the identified features identified for fatigue. In the case of joint pain, 7.0% had both the core and augmented features for joint pain. 4.2% had one of the core features for joint pain. 15.2% had one of the augmented features for joint pain. 73.6% had none of the features identified for joint pain.

**Table 2:** Patient characteristics of the augmented definition and the patient characteristics compared to the entire patient population.

	Patient No. (%)			
	Total Population n = 8,344	Dyspnea n = 484	Fatigue n = 398	Joint Pain n = 560
**Mean Age, years**	61.2 (19.6)	65.7 (16.2)	64.8 (17.1)	65.2 (16.2)
**Pre-COVID-19 Comorbidities**				
**Hypertension**	3215 (38.5)	304 (62.8)	254 (63.8)	353 (63.0)
**Diabetes**	1738 (20.8)	136 (28.1)	141 (35.4)	207 (37.0)
**Cardiovascular diseases**	2549 (30.5)	246 (50.8)	203 (51.0)	247 (44.1)
**Neurological diseases**	738 (8.8)	64 (13.2)	64 (16.1)	73 (13.0)
**Malignant tumor**	270 (3.2)	31 (6.4)	17 (4.3)	19 (3.4)
**Chronic pulmonary disease**	1303 (15.6)	139 (28.7)	108 (27.1)	160 (28.6)
**Chronic kidney disease**	1304 (15.6)	134 (27.7)	120 (30.2)	133 (23.8)

The clinical team reviewed a sampling of clinical notes for patients in healthcare system 1, belonging to each of the four categories, focusing on those notes written within the month of the structured data element of interest. Based on sampling and chart review, the patients with both the core and augmented features had the highest positive predictive value (PPV) of having the specific symptom for dyspnea at greater than three months after their incident COVID-19 date (73% [95% CI: 59–87] PPV). In the case of fatigue, patients with both a core and augmented feature had a 74% [95% CI: 60–88] PPV for the specific feature. In the case of joint pain, the augmented definition performed best with a core a 91% [95% CI: 82–100] PPV. See S6 Table for each group PPV. When the groups are combined, 25.0% (CI 95%: 5.5–48.4) of hospitalized patients have dyspnea, 12.3% (CI 95%: 6.7–17.9) have fatigue, and 14.3% (CI 95%: 10.0–18.5) have joint pain at 3 months.

The chronic conditions for the patients identified with the augmented definition were then analyzed. The mean ages for each of the three phenotypes was significantly greater than that of the total population (Table 2). Pre-existing hypertension was significantly more common among those with ongoing dyspnea (62.8%), fatigue (63.8%), and joint pain (63.0%), compared to the population of those hospitalized with COVID-19 (38.5%). Underlying diabetes was also more common with dyspnea (28.1%), fatigue (35.4%), and joint pain (37.0%), compared to the overall population (20.8%). Similar differences were seen among pre-existing cardiovascular disease, neurological disease, chronic pulmonary disease, and chronic kidney disease.

## Discussion

Previous studies have leveraged different techniques for identifying long COVID patients. Pfaff et al. used visits to the long COVID clinic as a proxy for long COVID [33]. However, access to a long COVID clinic remains uneven, and there may be specific patient interactions within a highly specialized healthcare system that enable such access. It is unclear whether the EHR features for identifying these patients would be generalizable to the broader population. As with the diagnosis code, this approach does not differentiate among the types of symptoms that one could develop with long COVID.

The Global Burden of Disease Long COVID Collaborators used a variety of different data sources including primary literature and relied heavily on the claims data from two separate networks [11]. However, the claims data analysis still fundamentally relied on a group of experts determining *a priori* a group of ICD codes that would define a symptom cluster of interest. As with any group using prior knowledge, their identified labels could miss certain patients that didn’t get that specific symptom code.

The value in our approach is that we enhance our initial definitions with a distributed learning approach to improve definitions so that they have a higher positive predictive value. These new definitions can then be used for understanding the true prevalence of the disease and analyzing the group of patients most likely suffering from the disease. Accurate tools for identifying such patients could enhance screening capabilities for the recruitment for clinical trials, improve the reliability of disease estimates, and allow for more accurate downstream cohort analysis. This includes the potential to detect rare associations and understand complex non-linear relationships.

The results of our analysis identify several important findings. Previous studies have shown that underlying comorbidities increase your likelihood of severe acute COVID-19 [34]. Pfaff et al. also suggested that greater comorbidities before acute COVID-19 contribute to greater likelihood of Long COVID [33]. Hanson et al. did not specifically look at comorbidities but they also identified that more severe COVID-19 infections were likely to lead to long COVID [11]. Our manuscript shows that underlying diseases was associated with increased likelihood of developing long COVID across all three symptom clusters studied. Even in the case of relatively less devastating chronic illness, like hypertension, there is an increased prevalence among those who went on to develop long COVID.

This finding has important implications for clinical care. Providers should consider increased vigilance when evaluating patients with multiple underlying comorbidities for the possibility they may have an increased likelihood of suffering from long COVID. It has long been known that patients with comorbidities are at increased risk of severe COVID [35]. Clinicians will need to continue their increased vigilance of patients with co-morbid conditions after the initial COVID-19 infection.

Our approach provides a robust and scalable framework for identifying patients with specific PASC subtypes in the EHR. Robustness of this study stems from its integration of clinical knowledge and data-driven discovery using distributed representation learning across multiple health systems, and validation through chart reviews by clinician experts. Scalability of this framework is based on its utilization of widely accessible structured EHR data, which can identify patients with the three PASC subtypes with reasonable accuracy. In the future, these augmented definitions could be applied in other healthcare systems to quickly ascertain persistent symptoms.

### Limitations

As with any EHR study, patients may be missed who go to a provider outside of the specific healthcare system and their ongoing symptoms might not be recorded in the EHR. However, each of the sites are large healthcare networks that include both primary care and tertiary academic medical centers. Additionally, since this study focused on patients hospitalized in three academic medical networks, there may be unique coding techniques among such centers compared to for-profit hospital systems. However, this is unlikely to be a significant limitation as the networks still include both large, specialized academic centers as well as smaller, community hospitals. The patients were still likely treated from a diverse array of providers. This study did only include hospitalized patients and therefore it is unknown how these findings would affect patients treated for their acute infection at home or those who were asymptomatic. Since only hospitalized patients were included, the population is older and sicker than the general population. Additionally, the chart review process was carried out by a single clinician, rather than multiple clinicians, which could introduce some bias. However, despite this limitation, the authors are unaware of other large epidemiological COVID-19 manuscripts that have used structured data and validated this with chart reviews.

## Supporting information

S1 Text 4CE membership. (DOCX)

S2 Text Association mining with MLHO. (DOC)

S1 Table Data dictionary for 4CE dataset. (DOC)

S2 Table Comorbidity grouping of Elixhauser comorbidities. (DOCX)

S3 Table Initial features definitions for long COVID created by clinical team. (DOCX)

S4 Table Complete list of digital features identified by the MHLO framework. (DOCX)

S5 Table AUC-ROC values for modeling the three designated PASC phenotypes in the three healthcare systems without the core features. (DOCX)

S6 Table Chart validation of the patients with core and augmented features and the associated positive predictive value from healthcare system 1. (DOCX)

S1 Fig Four separate groups identified by chart review. (TIFF)
