# Supplementary material for: A retrospective cohort analysis leveraging augmented intelligence to characterize long COVID in the electronic health record: A precision medicine framework
Source: PLOS Digit Health. 2023 Jul 25;2(7):e0000301. doi: 10.1371/journal.pdig.0000301 (PMC10368277; doi:10.1371/journal.pdig.0000301)
Supplement: S1 Text — (DOCX) [file pdig.0000301.s001.docx]

S1 Text. 4CE membership.

Dated June 26^th^, 2023

Vincent Benoit, Mélodie Bernaux, Romain Bey, Stéphane Bréant, Anita Burgun, Sylvie Cormont, Julien Dubiel, Martin Hilka, Judith Leblanc, Patricia Martel, Nina Orlova, Nicolas Paris, Elisa Salamanca, Arnaud Sandrin, Patricia Serre, Arnaud Serret-Larmande, Christel Daniel, Nicolas Griffon, Lorenzo Chiudinelli, Alberto Zambelli, Michele Beraghi, Mario Alessiani, Anna Alloni, Mauro Bucalo, Jayson Marwaha, Ashley Pfaff, Bertrand Moal, Guillaume Verdy, Sébastien Cossin, Romain Griffier, Vianney Jouhet, Krista Chen, Mohamad Daniar, Alon Geva, Kenneth Mandl, Piotr Sliz, Karen Olson, Emily Bucholz, Audrey Dionne, Jane Newburger, Paula Azevedo, Andrew South, Jason Moore, Batsal Devkota, Aaron Masino, Taha Mohseni Ahooyi, Nadir Yehya, Bruce Aronow, Surbhi Bhatnagar, Damien Leprovost, Andrew Vallejos, Robson Prudente, Danilo Amendola, Carlos Tadeu Neto, Janaina Santos, Marcelo Martins, Douglas Bell, Robert Follett, Douglas Murad, Fernando Sanz Vidorreta, Rachel Ramoni, Chuan Hong, Arthur Mensch, Antonio Bellasi, Sara Lozano-Zahonero, Adeline Makoudjou, Andrea Prunotto, Daniela Zöller, Christian Haverkamp, Raffaele Bruno, Paolo Sacchi, Valentina Zuccaro, Neil Sebire, John Booth, William Bryant, Richard Issitt, Daniel Key, Karyn Moshal, Mohsin Shah, Anastasia Spiridou, Anne Sophie Jannot, Paul Avillach, Brett Beaulieu-Jones, Clara-Lea Bonzel, Florence Bourgeois, Gabriel Brat, Nicholas Brown, Tianxi Cai, Priyam Das, Nils Gehlenborg, Alba Gutiérrez-Sacristán, Kenneth Huling, Mark Keller, Isaac Kohane, Sehi L'Yi, Anupama Maram, Sajad Mousavi, Thomas Naughton, James Norman, Nathan Palmer, Danielle Pillion, Zahra Shakeri Hossein Abad, Amelia Tan, Margaret Vella, Xuan Wang, Griffin Weber, William Yuan, Harrison Zhang, Molei Liu, Rui Duan, Larry Han, Xin Xiong, Adem Albayrak, Sadiqa Mahmood, Maryna Raskin, José Luis Bernal-Sobrino, Alvar Blanco-Martínez, Juan Luis Cruz-Bermúdez, Jaime Cruz-Rojo, Noelia García-Barrio, Cinta Moraleda, Miguel Pedrera-Jiménez, Víctor Quirós-González, Gustavo Roig-Domínguez, Pablo Rojo, Paula Rubio-Mayo, Pablo Serrano-Balazote, Ana Terriza-Torres, Antoine Neuraz, François Angoulvant, Michele Vitacca, Julien Champ, Silvano Bosari, Sara Pizzimenti, Luigia Scudeller, Derek Hazard, Patric Tippmann, Martin Wolkewitz, Marcos Minicucci, Marina Okoshi, Suzana Tanni, Luca Chiovato, Alberto Malovini, Valentina Tibollo, Maria Savino, Hossein Estiri, Chris Kennedy, Jeffrey Klann, Shawn Murphy, Zachary Strasser, Kavishwar Wagholikar, Siegbert Rieg, Andrew Atz, Jean Craig, Katie Kirchoff, Leslie Lenert, Jihad Obeid, Li Anthony, Yi-Ju Tseng, Kee Yuan Ngiam, Ne Hooi Will Loh, Bryce Tan, Byorn Tan, Scott Wong, Emma Toh, Robert Bradford, Meghan Hutch, Yuan Luo, Chengsheng Mao, L. Nelson Sanchez-Pinto, Emily Getzen, Loic Esteve, Jill-Jênn Vie, James Balshi, Aldo Carmona, Charles Sonday, Martin Boeker, Ian Krantz, Deanne Taylor, Fatima Ashraf, Emily Pfaff, Carlos Sáez, Detlef Kraska, Juergen Schuettler, Giuseppe Agapito, Mario Cannataro, Maria Mazzitelli, Marianna Milano, Carlo Torti, Enrico Trecarichi, Chiara Zucco, Thomas Ganslandt, Tobias Gradinger, Nandhini Santhanam, Lav Patel, James Cimino, Tiago Colicchio, Hans Prokosch, James Aaron, Jin Chen, Darren Henderson, Ramakanth Kavuluru, Shirley Fan, Lana Garmire, Bing He, Sarah Maidlow, Gilbert Omenn, Domenick Silvio, David Hanauer, Lemuel Waitman, Ashok Krishnamurthy, Giuseppe Albi, Riccardo Bellazzi, Arianna Dagliati, Emily Schriver, Trang Le, Qi Long, Jeffrey Morris, Danielle Mowery, Wanjiku Njoroge, John Holmes, Michele Morris, Malarkodi Jebathilagam Samayamuthu, Shyam Visweswaran, Zongqi Xia, Ye Ye, Jiyeon Son, Charlotte Caucheteux, Alexandre Gramfort, Olivier Grisel, Guillaume Lemaitre, Demian Wassermann, Gael Varoquaux, Kelly Cho, Yuk-Lam Ho, Petra Schubert, Scott DuVall, Kristine Lynch, and Brian Ostasiewski
