# Supplementary material for: A retrospective cohort analysis leveraging augmented intelligence to characterize long COVID in the electronic health record: A precision medicine framework
Source: PLOS Digit Health. 2023 Jul 25;2(7):e0000301. doi: 10.1371/journal.pdig.0000301 (PMC10368277; doi:10.1371/journal.pdig.0000301)
Supplement: S2 Text — (DOC) [file pdig.0000301.s002.doc]

**S2 Text - Association Mining with MLHO**

The MLHO algorithm uses a probabilistic approach to mining association rules, using an entropy-based algorithm, which provides a powerful tool for capturing non-linear associations in clinical data while also incorporating sparsity in identifying associations. The MLHO PheWAS implementation in the PascPhen package included the following steps:

1. 4CE data model is transformed to the MLHO input data model and the index date is set for all patients.
2. EHR data are time stamped based on the index data into pre-COVID (pre hospitalization), acute+ phase (0-90 days after hospitalization), and post-COVID (> 90 days after COVID-19 hospitalization).
3. Using the initial data elements, potential patients were identified with these specific symptoms after a SARS-2-CoV infection. A cohort is defined for each PASC sub-type if a core feature is present in a patient’s medical record for the first time 90 days or longer since hospitalization. Patients are labeled as positive for a phenotype if they meet the minimum criteria definition and negative if they do not meet the minimum criteria for the PASC subtype and have at least a follow up data point during the post-COVID time. A one year look back period before the initial SARS-2-CoV infection was used for each patient.
4. The core features are removed from the medical records and MLHO is applied to identify data elements during the post-COVID and acute+ phase that can predict the label for a given phenotype. MLHO (a) uses the minimize sparsity, maximize relevance (MSMR) dimensionality reduction algorithm that leverages joint mutual information and sparsity screening to (b) train gradient boosting machine classification models with 5-fold cross validation for (c) identifying EHR data elements that associate with a patient belonging to a given PASC sub-type cohort definition defined in step 3.

Step 4 was iterated five times (with a 80-20 train-test split and 5-fold cross validation) to compute MLHO confidence score, which quantifies the number of times a feature is identified as a predictor for a prediction/classification task, taking into account possible discrepancies between the direction of the association based on Odds Ratio (i.e., being a risk or protective factor).

The association mining process identified additional data elements which were recorded in the EHR data of patients labeled under each of the three PASC symptoms following hospitalization during acute COVID-19, where the initial list of codes for each symptom was removed. Each of the three healthcare systems ran MLHO and reported the identified representations along with the associated Odds Ratios and the classification performance metric, measured as the area under the receiver operating characteristics curve (AUROC). We used the recommended representations as MLHO features (see S3 Tab). The data-driven distributed learning was a computational augmentation of the initial expert-driven definitions for each PASC sub-phenotype.
