# Supplementary material for: A retrospective cohort analysis leveraging augmented intelligence to characterize long COVID in the electronic health record: A precision medicine framework
Source: PLOS Digit Health. 2023 Jul 25;2(7):e0000301. doi: 10.1371/journal.pdig.0000301 (PMC10368277; doi:10.1371/journal.pdig.0000301)
Supplement: S1 Table — (DOC) [file pdig.0000301.s003.doc]

S1 Table – Data dictionary for 4CE dataset.

| **Feature Types** | **Included in 4CE Data Set** |
| --- | --- |
| Diagnoses | Full set of ICD-9 and ICD-10 diagnoses |
| Procedure Types | Arterial Catheter, Bronchoscopy, CPR, Chest CT, ECMP, Emergency General Surgery, Emergency OBGYN, Emergency Orthopedics, Emergency Vascular Surgery, Renal Replacement, Supplemental Oxygen Other, Supplemental Oxygen Severe |
| Medications | ACE inhibitors, Angiotensin II receptor blockers, Antithrombotic agents, COVID-related antivrials, Diuretics, Aminoquinolines, Interleukin inhibitor, Interferon, Anesthesia-related medication, Cardiac-related medication |
| Laboratory Tests | Alanine aminotransferase, albumin, aspartate aminotransferase, total bilirubin, C-reactive protein, PaCO2, creatinine, ferritin, lactate dehydrogenase, PaO2, fibrinogen, procalcitonin, INR, D-Dimer (FEU), D-Dimer (DDU), cardiac troponin, prothrombin time, cardiac troponin, white blood cell count, lymphocyte count, neutrophil count, platelet count, blood gases, COVID positive test, COVID negative test |
