# Supplementary material for: A retrospective cohort analysis leveraging augmented intelligence to characterize long COVID in the electronic health record: A precision medicine framework
Source: PLOS Digit Health. 2023 Jul 25;2(7):e0000301. doi: 10.1371/journal.pdig.0000301 (PMC10368277; doi:10.1371/journal.pdig.0000301)
Supplement: S2 Table — (DOCX) [file pdig.0000301.s004.docx]

S2 Table- Comorbidity grouping of Elixhauser comorbidities.

| **Comorbidity** | **Elixhauser Comorbidities** |
| --- | --- |
| Hypertension | hypounc (uncomplicated hypertension), hypc (complicated hypertension) |
| Diabetes | diabunc (uncomplicated diabetes), diabc (complicated diabetes) |
| Cardiovascular diseases | chf (congestive heart failure), Carit (cardiac arrythmias), valv (valvular disease) |
| Neurological diseases | para (paralysis), ond (other neurological disorders) |
| Malignant tumor | metacanc (metastatic cancer) |
| Chronic pulmonary disease | cpd (chronic pulmonary disease) |
| Chronic kidney disease | rf (renal failure) |
