# Supplementary material for: A retrospective cohort analysis leveraging augmented intelligence to characterize long COVID in the electronic health record: A precision medicine framework
Source: PLOS Digit Health. 2023 Jul 25;2(7):e0000301. doi: 10.1371/journal.pdig.0000301 (PMC10368277; doi:10.1371/journal.pdig.0000301)
Supplement: S3 Table — (DOCX) [file pdig.0000301.s005.docx]

**S3 Table – Initial feature definitions for long COVID created by clinical team.**

| Phenotypes | Digital Biomarkers |
| --- | --- |
| Dyspnea | R06.0 Dyspnea  R06.00 Dyspnea, unspecified  R06.01 Orthopnea  R06.02 Shortness of breath  R06.03 Acute respiratory distress  R06.09 Other forms of dyspnea |
| Joint Pain | M25.50 Pain in unspecified joint  M25.511 Pain in right shoulder  M25.512 Pain in left shoulder  M25.519 Pain in unspecified shoulder  M25.521 Pain in right elbow  M25.522 Pain in left elbow  M25.529 Pain in unspecified elbow  M25.531 Pain in right wrist  M25.532 Pain in left wrist  M25.539 Pain in unspecified wrist  M25.541 Pain in joints of right hand  M25.542 Pain in joints of left hand  M25.549 Pain in joints of unspecified hand  M25.551 Pain in right hip  M25.552 Pain in left hip  M25.559 Pain in unspecified hip  M25.561 Pain in right knee  M25.562 Pain in left knee  M25.569 Pain in unspecified knee  M25.571 Pain in right ankle and joints of right foot  M25.572 Pain in left ankle and joints of left foot  M25.579 Pain in unspec. ankle and joints of unspec. foot |
| Fatigue | R530 Neoplastic (malignant) related fatigue  R53.1 Weakness  R53.81 Other malaise  R53.82 Chronic fatigue, unspecified  R53.83 Other fatigue |
