# Supplementary material for: A retrospective cohort analysis leveraging augmented intelligence to characterize long COVID in the electronic health record: A precision medicine framework
Source: PLOS Digit Health. 2023 Jul 25;2(7):e0000301. doi: 10.1371/journal.pdig.0000301 (PMC10368277; doi:10.1371/journal.pdig.0000301)
Supplement: S4 Table — (DOCX) [file pdig.0000301.s006.docx]

S4 Table – Complete list of digital features identified by the MHLO framework

| Phenotypes | Digital Biomarkers | Digital Biomarker Definition |
| --- | --- | --- |
| Dyspnea | DIAG-ICD10_Z8616_day90plus | Personal history of COVID-19 |
|  | COVID-TEST_covidU071_day90plus | COVID TEST |
|  | DIAG-ICD10_G4733_day90plus | Obstructive sleep apnea (adult) (pediatric) |
|  | DIAG-ICD10_I10_day90plus | Essential (primary) hypertension |
|  | DIAG-ICD10_R0902_day90plus | Hypoxemia |
|  | DIAG-ICD10_B948_day90plus | Sequelae of other specified infectious and parasitic diseases |
|  | COVID-TEST_covidneg_day90plus | COVID TEST |
|  | COVID-TEST_covidU071_day0to29 | COVID TEST |
|  | DIAG-ICD10_G629_day90plus | Polyneuropathy, unspecified |
|  | DIAG-ICD10_G7281_day90plus | Critical illness myopathy |
|  | DIAG-ICD10_I2510_day90plus | Atherosclerotic heart disease of native coronary artery without angina pectoris |
|  | DIAG-ICD10_I480_day90plus | Paroxysmal atrial fibrillation |
|  | DIAG-ICD10_I4891_day90plus | Unspecified atrial fibrillation |
|  | DIAG-ICD10_I509_day90plus | Heart failure, unspecified |
|  | DIAG-ICD10_I517_day90plus | Cardiomegaly |
|  | DIAG-ICD10_K219_day90plus | Gastro-esophageal reflux disease without esophagitis |
|  | LAB-LOINC_48065-7_day90plus | LOINC - D-dimer |
|  | LAB-LOINC_6598-7_day90plus | LOINC - Troponin T |
|  | LAB-LOINC_49563-0_day90plus | LOINC - Troponin I |
|  | LAB-LOINC_2276-4_day90plus | LOINC - Ferritin |
|  | MED-CLASS_DIURETIC_day90plus | MEDS Diuertic |
|  | MED-CLASS_ARB_day90plus | MEDS ARB |
|  | PROC-GROUP_CTChest_day90plus | PROCEDURE CT Chest |
|  | DIAG-ICD10_R05_day90plus | Cough |
|  | DIAG-ICD10_R079_day90plus | Chest pain, unspecified |
|  | DIAG-ICD10_R5381_day90plus | Other malaise |
|  | DIAG-ICD10_R918_day90plus | Other nonspecific abnormal finding of lung field |
|  | DIAG-ICD10_U099_day90plus | Post COVID-19 condition, unspecified |
|  | DIAG-ICD10_Z20822_day90plus | Contact with and (suspected) exposure to COVID-19 |
| Joint Pain | DIAG-ICD10_G8929_day90plus | Other chronic pain |
|  | DIAG-ICD10_I10_day90plus | Essential (primary) hypertension |
|  | DIAG-ICD10_K219_day90plus | Gastro-esophageal reflux disease without esophagitis |
|  | DIAG-ICD10_R5383_day90plus | Other fatigue |
|  | COVID-TEST_covidneg_day90plus | COVID test |
|  | DIAG-ICD10_E039_day90plus | Hypothyroidism, unspecified |
|  | DIAG-ICD10_E559_day90plus | Vitamin D deficiency, unspecified |
|  | DIAG-ICD10_E785_day90plus | Hyperlipidemia, unspecified |
|  | DIAG-ICD10_F17210_day90plus | Nicotine dependence |
|  | LAB-LOINC_2160-0_day90plus | LOINC Creatinine |
|  | LAB-LOINC_1988-5_day90plus | LOINC CRP |
|  | DIAG-ICD10_M170_day90plus | Bilateral primary osteoarthritis of knee |
|  | DIAG-ICD10_M1711_day90plus | Unilateral primary osteoarthritis, right knee |
|  | DIAG-ICD10_M545_day90plus | Low Back Pain |
|  | DIAG-ICD10_M7989_day90plus | Other specified soft tissue disorders |
|  | MED-CLASS_COAGA_day90plus | MEDS COAGA |
|  | MED-CLASS_ACEI_day90plus | MEDS ACEI |
|  | MED-CLASS_ARB_day90plus | MEDS ARB |
|  | DIAG-ICD10_R109_day90plus | Unspecified abdominal pain |
|  | DIAG-ICD10_R110_day90plus | Nausea |
|  | DIAG-ICD10_R2689_day90plus | Other abnormalities of gait and mobility |
|  | DIAG-ICD10_R42_day90plus | Dizziness and giddiness |
|  | DIAG-ICD10_R519_day90plus | Headache, unspecified |
|  | DIAG-ICD10_R52_day90plus | Pain, unspecified |
|  | DIAG-ICD10_R531_day90plus | Weakness |
|  | DIAG-ICD10_R918_day90plus | Other nonspecific abnormal finding of lung field |
|  | DIAG-ICD10_W19XXXA_day90plus | Unspecified fall, initial encounter |
|  | DIAG-ICD10_Y939_day90plus | Activity, unspecified |
|  | DIAG-ICD10_Z0000_day90plus | Encounter for general adult medical examination without abnormal findings |
|  | DIAG-ICD10_Z20822_day90plus | Contact with an (suspected) exposure to COVID-19 |
|  | DIAG-ICD10_Z23_day90plus | Encounter for immunization |
|  | DIAG-ICD10_Z4789_day90plus | Encounter for other orthopedic aftercare |
|  | DIAG-ICD10_Z789_day90plus | Other specified health status |
|  | DIAG-ICD10_Z8616_day90plus | Personal history of COVID-19 |
|  | DIAG-ICD10_Z87891_day90plus | Personal history of nicotine dependence |
|  | DIAG-ICD10_Z9189_day0to29 | Other specified personal risk factors, not elsewhere classified |
|  | DIAG-ICD10_Z9189_day30to59 | Other specified personal risk factors, not elsewhere classified |
|  | DIAG-ICD10_Z98890_day30to59 | Other specified postprocedural states |
|  | DIAG-ICD10_Z98890_day90plus | Other specified postprocedural states |
| Fatigue | DIAG-ICD10_Z8616_day90plus | Personal history of COVID-19 |
|  | DIAG-ICD10_E119_day90plus | Type 2 diabetes mellitus without complications |
|  | LAB-LOINC_2276-4_day90plus | LOINC Ferritin |
|  | DIAG-ICD10_Z20822_day90plus | Contact with and (suspected) exposure to COVID-19 |
|  | DIAG-ICD10_B948_day90plus | Sequelae of other specified infectious and parasitic diseases |
|  | COVID-TEST_covidneg_day90plus | COVID TEST |
|  | COVID-TEST_covidU071_day0to29 | COVID TEST |
|  | DIAG-ICD10_E039_day90plus | Hypothyroidism, unspecified |
|  | DIAG-ICD10_E782_day90plus | Mixed hyperlipidemia |
|  | DIAG-ICD10_F32A_day90plus | Depression, unspecified |
|  | DIAG-ICD10_F419_day90plus | Anxiety disorder, unspecified |
|  | DIAG-ICD10_G4733_day90plus | Obstructive sleep apnea (adult) (pediatric) |
|  | DIAG-ICD10_G7281_day90plus | Critical illness myopathy |
|  | DIAG-ICD10_G8929_day90plus | Other chronic pain |
|  | DIAG-ICD10_I10_day90plus | Essential (primary) hypertension |
|  | DIAG-ICD10_J189_day90plus | Pneumonia, unspecified organism |
|  | LAB-LOINC_2160-0_dayN14toN1 | LOINC Creatinine |
|  | LAB-LOINC_49563-0_day90plus | LOINC Troponin I |
|  | LAB-LOINC_2160-0_day90plus | LOINC Creatinine |
|  | LAB-LOINC_6598-7_day90plus | LOINC Troponin T |
|  | DIAG-ICD10_M549_day90plus | Dorsalgia, unspecified |
|  | MED-CLASS_ARB_day90plus | MEDS ARB |
|  | PROC-GROUP_CTChest_day60to89 | PROCEDURE CT Chest |
|  | DIAG-ICD10_R0600_day90plus | Dyspnea, unspecified |
|  | DIAG-ICD10_R262_day90plus | Difficulty in walking, not elsewhere classified |
|  | DIAG-ICD10_U099_day90plus | Post COVID-19 condition, unspecified |
|  | DIAG-ICD10_Z87891_day90plus | Personal history of nicotine dependence |
|  | DIAG-ICD10_Z9049_day90plus | Acquired absence of other specified parts of digestive tract |
