# Supplementary material for: A retrospective cohort analysis leveraging augmented intelligence to characterize long COVID in the electronic health record: A precision medicine framework
Source: PLOS Digit Health. 2023 Jul 25;2(7):e0000301. doi: 10.1371/journal.pdig.0000301 (PMC10368277; doi:10.1371/journal.pdig.0000301)
Supplement: S5 Table — (DOCX) [file pdig.0000301.s007.docx]

S5 Table - AUC-ROC values for modeling the three designated PASC phenotypes in the three healthcare systems without the core features.

| **PASC phenotype** | **Healthcare System 1** | **Healthcare System 2** | **Healthcare System 3** |
| --- | --- | --- | --- |
| **Dyspnea** | 0·91 [0·89 - 0·91] | 0·81 [0·81 - 0·82] | 0·86 [ 0·85 - 0·86] |
| **Fatigue** | 0·90 [0·89 - 0·91] | 0·85 [0·85 - 0·86] | 0·84 [ 0·84 - 0·84] |
| **Joint Pain** | 0·78 [0.77 - 0·80]* | 0·84 [0·84-0·84] | 0·82 [0·82 - 0·83] |

* Area under the Receiver Operative Characteristics Curve (AUROC) and 95% Confidence Intervals

** the AUROCs were obtained by training classification models against data where labels indicated a cohort assignment (or lack thereof) to a particular long COVID phenotype.
