# Supplementary material for: A retrospective cohort analysis leveraging augmented intelligence to characterize long COVID in the electronic health record: A precision medicine framework
Source: PLOS Digit Health. 2023 Jul 25;2(7):e0000301. doi: 10.1371/journal.pdig.0000301 (PMC10368277; doi:10.1371/journal.pdig.0000301)
Supplement: S6 Table — (DOCX) [file pdig.0000301.s008.docx]

S6 Table. Chart validation of the patients with core and augmented features and the associated positive predictive value from healthcare system 1.

|  | **Dyspnea** | | **Fatigue** | | **Joint Pain** | |
| --- | --- | --- | --- | --- | --- | --- |
|  | **Pop.** | **PPV** | **Pop.** | **PPV** | **Pop.** | **PPV** |
| **Patients with core and augmented data elements** | 398 / 8,344 (4.8 %) | 0.73  [0.59 - 0.87] | 484 / 8,344 (5.8 %) | 0.74  [0.60 - 0.88] | 560 / 8,344 (6.7 %) | 0.91  [0.82 - 1.00] |
| **Patients with core data elements exclusive** | 259 / 8,344  (3.1 %) | 0.49  [0.34 - 0.64] | 141 / 8,344 (1.7 %) | 0.71  [0.58 - 0.84] | 339 / 8,344  (4.1 %) | 0.74  [0.60 - 0.88] |
| **Patients with augmented data elements exclusive** | 1328 / 8,344  (15.9 %) | 0.30  [0.10 - 0.50] | 1892 / 8,344  (22.7 %) | 0.30  [0.10 - 0.50] | 1,225 / 8,344  (14.7 %) | 0.35  [0.14 - 0.56] |
| **Patients with neither core nor augmented features** | 6,024 / 8,344  (76.2 %) | 0.20  [0.00 - 0.45] | 5,532 / 8,344  (69.8 %) | 0.00 | 5,925 / 8,344  (74.5 %) | 0.00 |
