# Supplementary figures and images for: A retrospective cohort analysis leveraging augmented intelligence to characterize long COVID in the electronic health record: A precision medicine framework
Source: PLOS Digit Health. 2023 Jul 25;2(7):e0000301. doi: 10.1371/journal.pdig.0000301 (PMC10368277; doi:10.1371/journal.pdig.0000301)

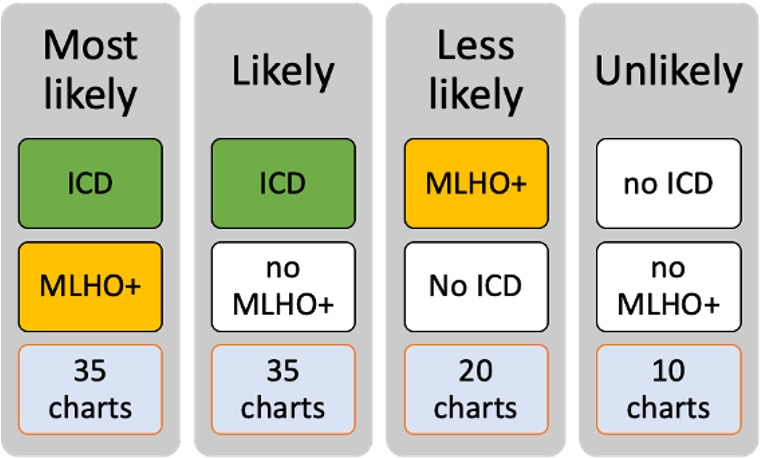

Supplement: S1 Fig — (TIFF) [file pdig.0000301.s009.tiff]
